# SPRENO: a BioC module for identifying organism terms in figure captions

**DOI:** 10.1093/database/bay048

**Published:** 2018-06-03

**Authors:** Hong-Jie Dai, Onkar Singh

**Affiliations:** 1 Department of Computer Science and Information Engineering, National Taitung University, 369, Sec. 2, University Rd., Taitung, Taiwan, R.O.C; 2 Interdisciplinary Program of Green and Information Technology, National Taitung University, 369, Sec. 2, University Rd., Taitung, Taiwan, R.O.C; 3 Bioinformatics Program, Taiwan International Graduate Program, Institute of Information Science, Academia Sinica, 128 Academia Road, Section 2, Nankang, Taipei, Taiwan, R.O.C; 4 Institute of Biomedical Informatics, National Yang-Ming University, No. 155, Sec. 2, Linong Street, Taipei, 112 Taiwan, R.O.C

## Abstract

Recent advances in biological research reveal that the majority of the experiments strive for comprehensive exploration of the biological system rather than targeting specific biological entities. The qualitative and quantitative findings of the investigations are often exclusively available in the form of figures in published papers. There is no denying that such findings have been instrumental in intensive understanding of biological processes and pathways. However, data as such is unacknowledged by machines as the descriptions in the figure captions comprise of sumptuous information in an ambiguous manner. The abbreviated term ‘SIN’ exemplifies such issue as it may stand for Sindbis virus or the sex-lethal interactor gene (*Drosophila melanogaster*). To overcome this ambiguity, entities should be identified by linking them to the respective entries in notable biological databases. Among all entity types, the task of identifying species plays a pivotal role in disambiguating related entities in the text. In this study, we present our species identification tool SPRENO (Species Recognition and Normalization), which is established for recognizing organism terms mentioned in figure captions and linking them to the NCBI taxonomy database by exploiting the contextual information from both the figure caption and the corresponding full text. To determine the ID of ambiguous organism mentions, two disambiguation methods have been developed. One is based on the majority rule to select the ID that has been successfully linked to previously mentioned organism terms. The other is a convolutional neural network (CNN) model trained by learning both the context and the distance information of the target organism mention. As a system based on the majority rule, SPRENO was one of the top-ranked systems in the BioCreative VI BioID track and achieved micro *F*-scores of 0.776 (entity recognition) and 0.755 (entity normalization) on the official test set, respectively. Additionally, the SPRENO-CNN exhibited better precisions with lower recalls and *F*-scores (0.720/0.711 for entity recognition/normalization). SPRENO is freely available at https://bigodatamining.github.io/software/201801/.

Database URL: https://bigodatamining.github.io/software/201801/

## Introduction

To facilitate a better understanding of the fundamental life processes, biological research nowadays tends to explore and perceive the biological system in its entirety rather than focusing on specific biological entities. Most of the observations resulting from these hypothesis-driven researches are exclusively available in the form of figures in published papers to help readers understand them in an effortless manner. Nevertheless, reviewing all of them manually is time-consuming and unrealistic due to the overwhelming amount of literatures available in PubMed. Therefore, in spite of their importance in apprehending biological processes and the mechanism of human diseases, the nuggets of information enclosed within the figures and their captions were hardly ever exploited since there were no effective tools or platforms that allow life scientists to browse and compare the scientific results presented in the figures of all related publications. In light of this, Liechti *et al.* (1) recently announced the initiative of the SourceData platform which can link related figures among various papers together to form a searchable knowledge graph. However, it requires the expertise of life science and the bio-curators’ effort to manually identify biomedical entities in the text and link them to their corresponding database entries. This process is labor-intensive but indispensable to ensure the quality of the data. Thus, it is essential to develop new methods and tools to reduce the time and effort bio-curators spent on recognizing entities in figure captions and associating them with their corresponding database IDs (2).

**Figure 1.**
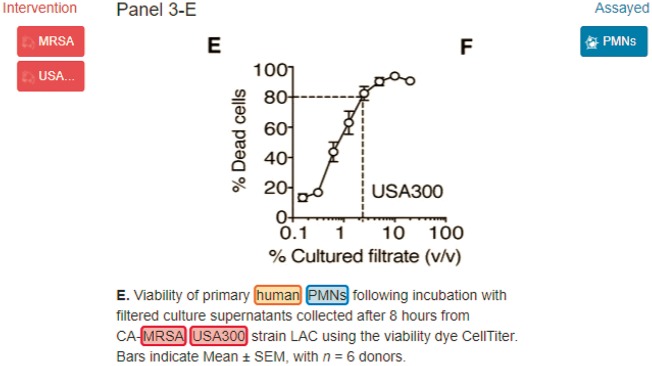
MRSA, USA300 and human are organism terms which should be linked to NCBI Tax: 1280, NCBI Tax: 367830 and NCBI Tax: 9606, respectively.

The Bio-ID track of BioCreative VI provided a dataset annotated by the SourceData curators with several types of biomedical entities including organisms that exist in the figure captions and their corresponding database IDs. Figure 1 displays an example of the annotations created by the SourceData curators. The recognition of organism helps curators or machines disambiguate the recognition of other bio-entities such as mutations, proteins or genes (3, 4). In addition, it enables users to access relevant subsets of publications based on species-specific queries.

Considering the importance of identifying organism terms, we extended our previous work (5) developed for recognizing species terms in abstracts to distinguish organism terms in figure captions. In comparison to the recognition of species terms in abstracts or even full texts, which have already been studied in previous works (6, 7), the process of identifying terms described in figure captions is more challenging owing to the considerable uncertainties resulting from the use of abbreviations of species names and the use of common English names instead of Latin names to refer to an organism. The ambiguous nature of organism terms recognized in figure captions prompts us to develop strategies exploiting information from full text to resolve the ambiguities. In addition, the frequent use of strain terms in the figure captions also makes the task even more challenging. Authors usually employ specialized terms of strains/models in figure captions to describe their experimental observations. For example, the terms of inbred strains of the mouse include C57BL/6J, R6/2, and DBA/2J. Strains are particularly difficult to detect because they do not follow any systematic nomenclature and most of them have no records in the NCBI taxonomy database. Aside from the organism tagger developed by Naderi *et al.* (8), strain mentions cannot be distinguished by most of the current openly available organism recognition tools.

In view of this concern, a new species recognition tool SPRENO (Species Recognition and Normalization) is introduced in this work. The lexicon used by our previous species recognition tool (5) developed for the BioCreative V BioC task (9) was expanded by including organism terms and terms referring to strains or models. We also developed new algorithms optimized for normalizing organism terms mentioned in figure captions by considering both the resident text and the corresponding full text. Ambiguous organism terms are disambiguated by the majority rule and a convolutional neural network (CNN) model, and their performances were investigated on the dataset provided by the Bio-ID track.

## Materials and methods

### Organism identification approach

**Figure 2.**
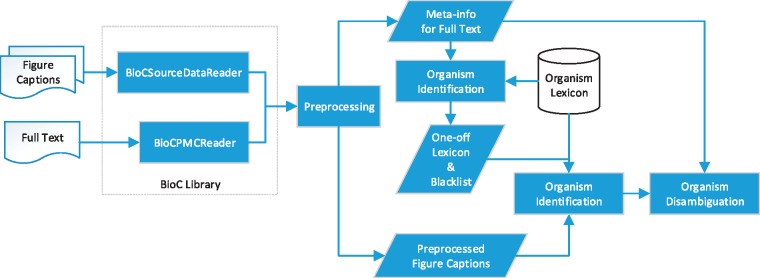
Workflow of SPRENO.

Motivated by the ambiguous nature of organism terms in figure captions, we extended the multistage organism identification algorithm developed in our previous work (5) to capitalize on the information collected from full text to resolve the ambiguities. The algorithm processes the content of the given full text prior to analysing the target figure caption since the full names of the abbreviated terms are usually defined in sections like the Introduction. Recognizing these names in advance can reduce the ambiguity of the frequently used abbreviations in figure captions accordingly. Figure 2 demonstrates the workflow of SPRENO.

Our previous BioC library (https://www.nuget.org/packages/NTTU.BigODM.Bio.BioC) was expanded to support the processing of figure captions represented in the BioC format defined by the Bio-ID task. In order to exploit the information from full texts, a new BioC reader for PMC has been implemented. The expanded library managed several span errors of annotations observed in the SourceData dataset owing to the Unicode encoding of special characters like ‘\u03B1’, ‘\u03B2’ and ‘\u25A0’. When one invokes SPRENO to identify organisms mentioned in a figure caption, the newly developed library is used to simultaneously load the figure caption and its corresponding full-text article. Both the contents of the full text and the figure caption were preprocessed to detect sentence boundaries, tokens, part-of-speech (PoS) tags and full name–abbreviation pairs. In this work, LingPipe (10) was employed to detect sentence boundaries, GENIA tagger (11) was used for tokenization and PoS tagging, and ExtractAbbrev (12) was utilized for recognizing full name–abbreviation pairs. Information provided by these tools is stored in a data structure called meta-info to supply the algorithm with necessary information collected from the full text for disambiguation.

Following the idea of the winning methodology in the BioCreative II.5 gene normalization task (13), the algorithm begins with sections with more abundant information when processing the full text, whereas sections with less information were assigned a lower priority during the processing to assist organism-term identification. In respect of our objective, sections with abundant information are those that are most likely to describe an organism’s full name. Therefore, it is best to start with the Introduction section as this is the section in which the authors present the organisms of interest for the first time, providing their full names followed by abbreviations used in the manuscript thereafter. Hence, for a given full-text article, the algorithm executes the matching procedure to identify organism terms in the order of the Introduction section, followed by the abstract and the other sections with the lexicon compiled for the task. Details of the compiled lexicon are delineated in the next section.

During the matching process, if the algorithm observes a full name–abbreviation pair, the base form of the full name is first matched with the lexicon. If the full name is considered to be an organism term, both the name and its abbreviation are added to the meta-info as additional organism terms for matching. Otherwise, the pair is blacklisted. The algorithm utilizes the compiled lexicon along with the additional terms listed in the meta-data to scan the entire article for organism mentions. After identifying all of the organism-term candidates, the PoS information is used to filter out false-positive cases such as candidates with the PoS as a verb.

### Lexicon expanded with terms of strain/model

The lexicon compiled in our previous work (5) was augmented to include terms indicative of organisms, common terms such as embryos and seedlings, and names of strains/model organisms for matching. Table 1 summarizes the resources used in this work. The compiled lexicon was further enhanced by adding the base form of each collected term generated by the GENIA tagger.

**Table 1.** Resources for strain/model terms

Source	Organism
http://www.findmice.org/repository	Mouse
http://www.informatics.jax.org/downloads/reports/index.html#strain	Mouse
http://www.criver.com/find-a-model	Mouse
http://gcm.wfcc.info/speciesPage.jsp? strain_name=Lactobacillus%20acidophilus#specTopgcm.wfcc.info	Lactobacillus acidophilus
https://gold.jgi.doe.gov/organisms? Organism.Domain=BACTERIAL&Organism.Type%20Strain=Yes&Organism.Active=Yes	Bacteria
https://byo.com/resources/yeast	Yeast

Each entry of the lexicon contains a NCBI taxonomy ID and its possible organism synonyms. Each character of the organism terms listed in the lexicon and the meta-info associated with the given figure were represented as a vertex to generate directed acyclic word graphs. When processing a figure caption, the matching algorithm matches the text with the generated graphs to recognize the corresponding entities. The algorithm was implemented to perform partial matching by allowing a mismatch with a length of three characters on the compiled word graphs. If a matched organism term is linked with more than one ID, the recognized entity is considered ambiguous and requires the application of the disambiguation method described in the next section to determine the correct ID.

### Disambiguation approach

In order to reduce the ambiguity of the recognized organism terms in figure captions, two disambiguation methods were applied. The first is a majority rule-based approach using the pre-linked information recorded in the meta-info for each article to disambiguate an ambiguous term. When processing a figure caption, whenever SPRENO encounters an organism term associated with more than one ID, the system checks the meta-info associated with the corresponding source article for the number of times these IDs have been successfully linked in that article. The ambiguous term will be assigned with the ID with the most pre-linked occurrences based on the property of identity transitivity (14). It is worth noting that the approach can benefit from the processing order of the multistage algorithm (13) as the order can result in a more accurate estimation of the number of pre-linked IDs for an ambiguous term.

**Figure 3.**
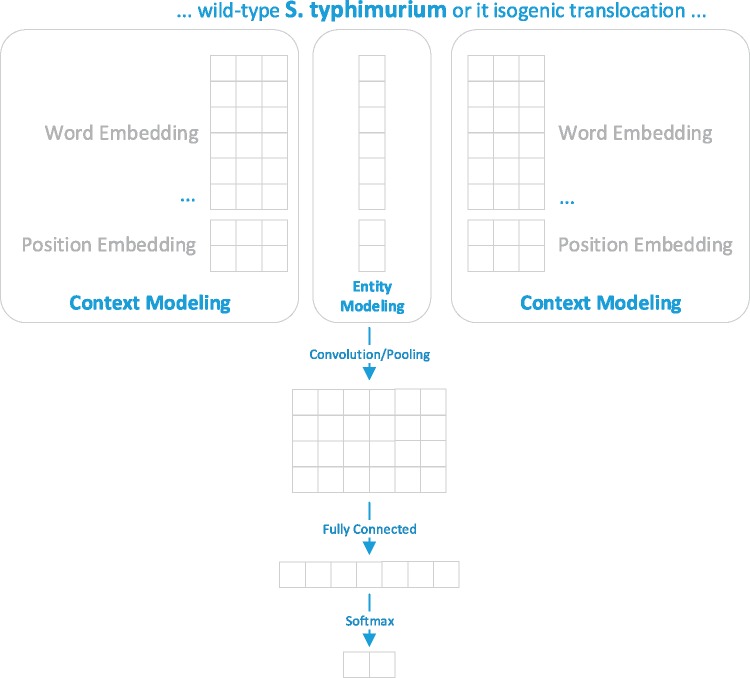
The developed CNN model for organism disambiguation. For the target mention ‘S. typhimurium’, whose linked NCBI Taxonomy ID is 90371, the context is represented as {…, wild/−1, S. typhimurium/0, or/1, its/2, …} and the entity is represented as its scientific name (i.e. Salmonella enterica subsp. enterica serovar Typhimurium).

The second disambiguation method is a machine learning method based on CNN. The disambiguation problem was formulated as a binary classification task and the training set was generated based on the outcome of the developed matching method. The training set included successfully linked terms and ambiguous terms along with their candidate IDs, as well as their surrounding context in the figure captions. Figure 3 presents an overview of the developed CNN model for organism disambiguation.

As shown in Figure 3, the input of our model includes the term of the mention (*S. typhimurium*), the surrounding context of the mention (‘… wild-type’ and ‘or its isogenic…’) and its candidate record (i.e. the scientific name ‘Salmonella enterica subsp. enterica serovar Typhimurium’) from the NCBI taxonomy database. The output is the probability distribution over two possible outcomes {yes, no}. The model relied on information conveyed by the context modeling and the entity modeling for disambiguating a target organism mention. The context modeling represents the surrounding context by (i) word representations for all surrounding words in a figure caption and (ii) the position representations which capture the distance between a context word and the target mention based on the consideration that a closer context word may be more informative than a further one (15). As for entity modeling, the target mention is represented as the official symbol recorded in the Taxonomy database.

In this work, the pre-trained PubMed word embedding with a dimension of 200 released by Moen *et al.* (16) was used for representing the words. A special word ‘OOV’ with a randomly initialized small weight value was added into the vocabulary generated from the training set to represent the out-of-vocabulary words which may appear in the test set. The learning rate and the window size of CNN were empirically set as 0.01 and 2, respectively.

### Common term recognition and normalization

**Figure 4.**
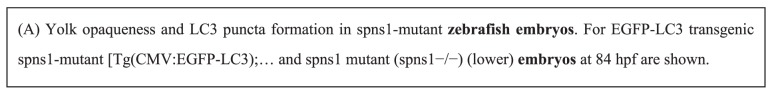
An example illustrating the common term (embryos) and its related species (zebrafish).

It was observed in the Bio-ID corpus that curators tend to annotate common terms like larvae and embryos for multiple organisms depending on the contextual information described in the article. Take Figure 4 as an example, in which the authors specified that they used zebrafish embryos when the common term first appeared in the figure caption. Consequently, the term ‘embryo(s)’ found in the caption and the figure captions thereafter should refer to the zebrafish embryo unless there are additional indications. It is noteworthy that the same term may be used to refer to different species within different articles. For example, based on our analysis of the training set, the term ‘embryo(s)’ may refer to that of the zebrafish (Taxonomy ID: 7955), mouse (Taxonomy ID: 10090), roundworm (Taxonomy ID: 6239) and chicken (Taxonomy ID: 9031).

We examined the training set of the Bio-ID corpus to collect all possible grounding IDs for the three common terms listed in Table 2. When processing a given article following the multistage algorithm, if we detect an occurrence of the common terms, the algorithm was designed to list all possible IDs related to the term as candidates. Among these candidates, the one with the most pre-linked occurrences will be selected as the final ID for the term.

**Table 2.** Common organism terms observed in the training set of the Bio-ID corpus

Common term	Possible taxonomy IDs
Larva	7227, 7955, 6239
Embryo	10 090, 7955, 6239, 9031
Seedling	3915, 3702

## Results

### Dataset

The corpus released in the BioC format by the BioCreative VI Bio-ID track (2) was used to evaluate the performance of SPRENO. The corpus contains annotations for organisms, genes, proteins, microRNAs, small molecules, cellular components, cell types, cell lines, tissues and organs mentioned in the figure panel captions from SourceData. The text may consist of discontinuous text derived from the figure captions, and it may also include legends of several figure panels. The dataset consists of 7960 and 2586 annotations of organism entities for the training and the test sets, respectively. Table 3 displays the statistics for the organism annotations in the corpus.

**Table 3.** Statistics of the organism annotations in the Bio-ID corpus

Dataset	# of articles	# of figure captions	# of annotations	# of unique IDs
Training set	570	13 576	7960	158
Test set	196	1458	2586	73

### Evaluation metrics

The evaluation metrics including micro-average precision (*P*), recall (*R*) and *F*-measure (*F*) were used to report the performance of SPRENO at both the mention and normalization level. At the mention level, the spans of the recognized entities were compared with the manually annotated ones. By contrast, at the normalized ID level; the set of unique IDs provided by the system for a caption was compared to the reference set of IDs in the corpus. The mention level organism annotations were further divided into two classes. The first class consists of annotations which are linked to the NCBI taxonomy database. For example, in Figure 1, ‘human’ is annotated with ‘NCBI Tax: 9606’. The second class comprises annotations that are associated with the organism type but not linked to the NCBI taxonomy database. For instance, in the text ‘Ubc9 variants were expressed in bacteria’, the term ‘acteria’ is tagged as ‘organism: bacteria’ to indicate bacteria as an organism, but it was not linked to a specific taxonomy ID.

### Official results in the Bio-ID track

We submitted three runs to assess the performance of the proposed organism identification method for recognizing and normalizing organism terms mentioned in figure captions. For the first run, the majority rule-based disambiguation method utilizing full-text information was applied. A threshold was set at two to filter out organism names that were matched with more than two IDs after the disambiguation process. In the second run, the threshold was increased to 10, that is organism mentions having >10 IDs were ruled out. Subsequently, the developed CNN model was employed to select the ID with the highest likelihood. Note that under circumstances in which a candidate organism is only associated with one ID after matching, the candidate and its ID were still processed by the CNN model to determine if they should be discarded. Finally, in order to investigate the effect of the compiled lexicon and the generalize ability of the developed CNN model, the third run was performed in which we used the lexicon compiled by Pafilis *et al.* (7) for their species/organism recognition tool along with the CNN-based disambiguation. The threshold for the last run was set to infinite so that no IDs were excluded. Therefore, SPRENO completely relied on our CNN model to determine the best ID for a candidate organism.

Table 4 demonstrates the official results on the test set of the Bio-ID track at the mention level with two different matching criteria. The strict criterion demands exact span matching, meaning that the predicted span needs to be exactly matched with that of the reference gold standard. By contrast, the overlap criterion allows the overlapping of the predictions with the reference annotations. As shown in Table 4, the first run achieved the best *R* and *F*-scores, while the second run achieved a better precision under both matching criteria. The third run was endowed with the worst PRF-scores. Table 4 also includes the results of the other two top-ranked systems developed by Sheng *et al.* (17) and Kaewphan *et al.* (18). Our best run ranked second and third under the strict and overlap criteria, respectively.

**Table 4.** Official organism recognition results on the test set

Configuration	Criterion	*P*	*R*	*F*
**Run 1**	Strict	0.663	**0.874**	**0.754**
	Overlap	0.683	**0.900**	**0.776**
**Run 2**	Strict	**0.671**	0.731	0.699
	Overlap	**0.690**	0.752	0.720
**Run 3**	Strict	0.516	0.251	0.337
	Overlap	0.536	0.260	0.350
**Sheng, Miller *et al.* (**17**)**	Strict	0.746	0.715	0.730
	Overlap	0.814	0.780	0.796
**Kaewphan, Mehryary *et al.* (**18**)**	Strict	**0.860**	**0.809**	**0.834**
	Overlap	**0.878**	**0.826**	**0.852**

Table 5 presents the official organism normalization results on the test set. The majority rule-based disambiguation method (Run 1) achieved the highest micro-*F*-score of 0.756, which outperformed the other two top-ranked systems developed by Sheng *et al.* and Kaewphan *et al.* by 0.027 and 0.089, respectively. By examining the normalization results of Run 1 and 2, we observed that the developed CNN-based disambiguation method is competent in distinguishing and removing false positive cases. Even if the candidate ID pool of Run 2 is larger than that of Run 1 due to the threshold setting, Run 2 still acquired a better precision. On the other hand, owing to the low recall rate of 0.476 of Pafilis *et al.*’s tool on the Bio-ID training set as reported by one of the participating teams (19), we did not filter out any IDs in the third run. However, it still obtained the lowest recall and the worst PF-scores. The dramatically low recall of Run 3 compared to the other runs indicates the necessity of compiling a new lexicon for the Bio-ID track, which will be further discussed in the following ‘Comparison with Other Participating Teams in the Bio-ID Track’ section. In addition, we noticed that the PRF-scores of Run 3 were lower than that reported in Chang *et al.*’s work (19). This may be partly due to the training set of the compiled CNN model that was generated based on our lexicon.

**Table 5.** Official organism normalization results on the test set

Configuration	*P*	*R*	*F*
**Run 1**	0.660	**0.883**	**0.756**
**Run 2**	**0.668**	0.760	0.711
**Run 3**	0.526	0.327	0.403
**Sheng, Miller *et al.* (**17**)**	**0.772**	**0.691**	**0.729**
**Kaewphan, Mehryary *et al.* (**18**)**	0.668	0.667	0.667

## Discussion

### Does full-text information really matter?

**Figure 5.**
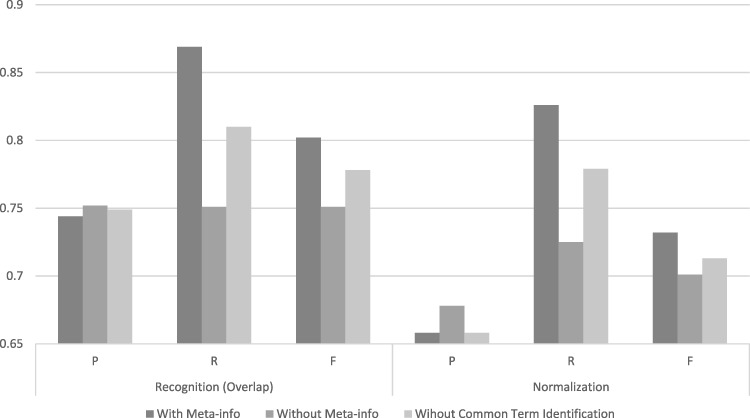
Performance comparison on the training set with or without using full-text information and common term identification.

One distinguishable feature of SPRENO is the use of meta-info from full text to improve the performance in recognizing and linking organism terms mentioned in figure captions. Figure 5 illustrates the advantage of exploiting full text information in SPRENO on the training set of the Bio-ID corpus. The results of three different configurations were depicted:

1. With Meta-info: The meta-info collected from full text was adapted by SPRENO.
2. Without Meta-info: SPRENO directly processes the text of a figure caption without using the information from full text.
3. Without Common Term Identification: This configuration intends to demonstrate the effectiveness of the method used for recognizing and normalizing common terms described in the Common Term Recognition and Normalization section. Note that the meta-info was used although the common term identification method was not applied in this configuration.

The configuration with meta-info had noticeably better recalls which resulted in the best *F*-scores in both the recognition and normalization tasks. The precisions were slightly higher when the meta-info or the common term identification method was not used. However, the recall is significantly reduced by 0.12 and 0.10 in the recognition and normalization tasks, respectively.

**Figure 6.**
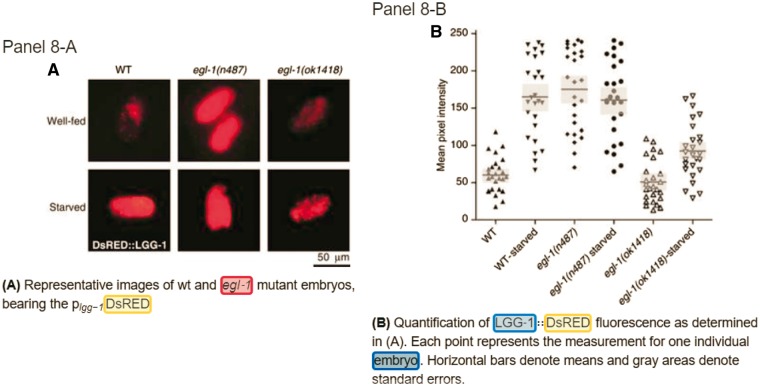
Annotations for Figure 8 of the paper PMC 186890 from SourceData (Refer to the link http://smartfigures.net/article/smartfigure/10.1038/sj.emboj.7601689/index/8).

An example from the paper PMC 1868901 is used to illustrate how the meta-info along with the proposed common term identification method can be used to improve the results of normalization. Figure 6 displays the annotated results on SourceData for panel A and B of Figure 8. As shown in panel 8-B, the caption contains a common term ‘embryo’, and not even a curator with related expertise can confirm the identity of this term merely based on the context of this caption. However, if we read through the content of the section describing this figure in the original paper (refer to the section named ‘Regulation of autophagy by the BH3-only protein EGL-1 in *C. elegans*’ in the paper PMC1868901), the descriptions ‘EGL-1 is the sole pro-apoptotic BH3-only protein in **C. elegans** and is required for developmental cell death in this **nematode**.’ and ‘…, Starvation strongly induced autophagy, and this induction was blunted in *egl-1*-deficient **nematode embryos**. In contrast, by starvation (Figure 8) (https://www.ncbi.nlm.nih.gov/pmc/articles/PMC1868901/figure/f8/).’ substantiate the fact that the embryos mentioned in the figure caption should belong to the nematode *C. elegans* (NCBI taxon: 6239). This case exemplifies the importance of full text information and the effectiveness of using meta-info and common term identification.

The reduction in the precision of our approach may be attributed to the incomprehensive nature of the Bio-ID corpus. We examined the corpus and found that not all organism terms within the figure captions were annotated. Take panel 8-A shown in Figure 6 as an example. Similar to panel 8-B, the caption also contains the term ‘embryos’, but SourceData did not provide an annotation for it. SPRENO equipped with the meta-info and common term identification will recognize the term and link it to NCBI taxon 6239, resulting in a false positive case.

### Distribution of different organism entity types observed in figure captions

**Figure 7.**
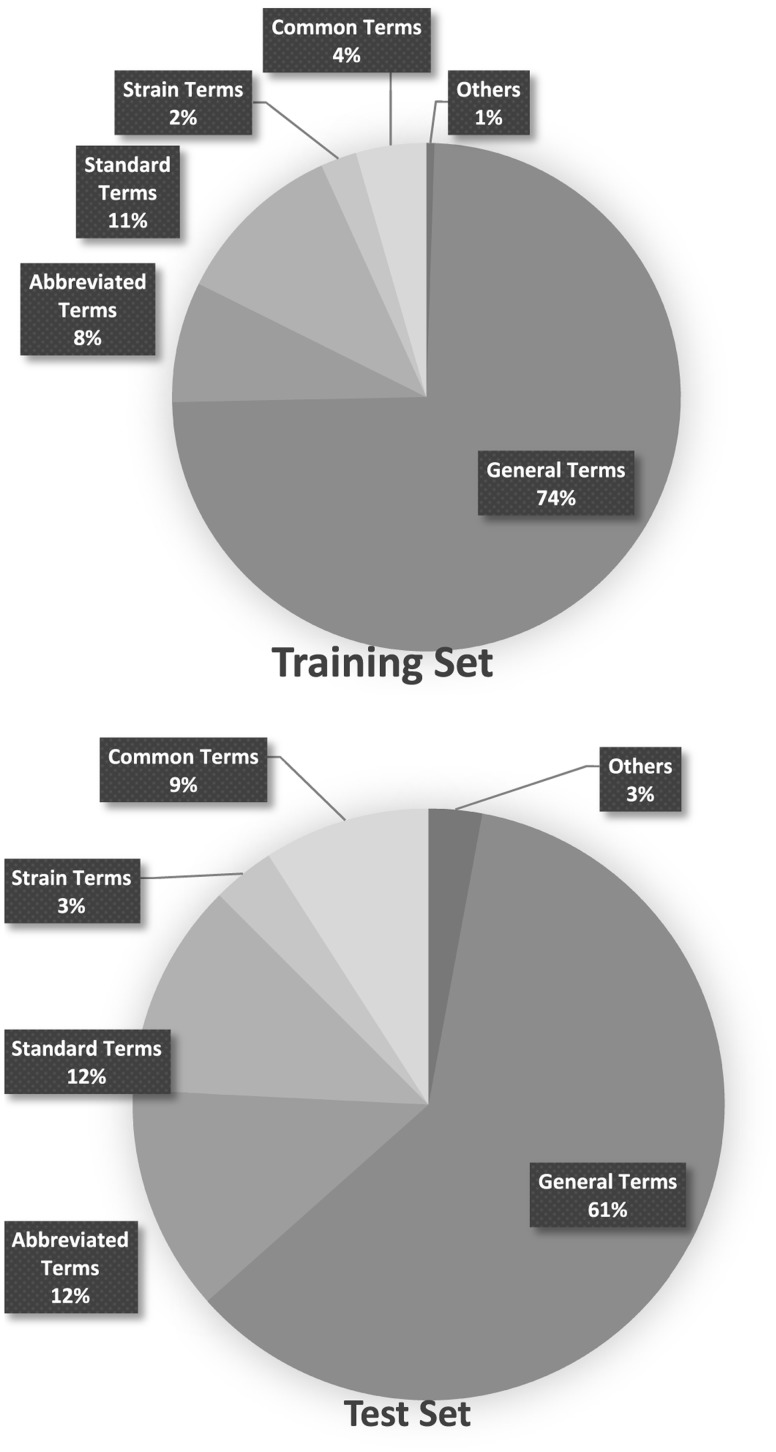
Distribution of the types of organism names in the Training and Test set.

The organism annotations provided in the Bio-ID corpus were manually analysed and categorized into the five types listed in Table 6. Graphs in Figure 7 shows the distribution of these types in both the training and test sets.

**Table 6.** Types of the organism name

Type	Description	Example
General terms	Following the definition from our previous work (5), general terms include terms that can be used as evidence to determine the organism of the co-occurring bio-entity.	Rat, fetus, mouse, human
Common terms	Terms that can be referred to various organisms depending on the contextual information.	Larva, embryo, seedling
Strain terms	Strains used in laboratory experiments.	C57BL/6, BJ3505, PR8
Abbreviated terms	Abbreviations that refer to organisms/species.	GBS, AAV, LV
Standard terms	Terms recorded in the Taxonomy Database.	Zebrafish, S. cerevisiae, E. coli
Others	Terms that cannot be categorized into the types above.	16-cell stage, zygote

The information provided in Figure 7 indicates that general terms, in combination with standard terms and abbreviated terms, make up the majority of the organism names found in figure captions. Abbreviated terms, however, contributed to most of the false negative cases of SPRENO, even though the full name–abbreviation pairs recorded in the meta-info have been exploited to address the issue caused by abbreviations. These error cases are expected to be solved with the use of additional lexicon resources. For example, LV, which should be linked to *Lentivirus* (NCBI taxon: 11646), was mentioned in several figure captions of the article PMC 5048368. It first appeared in the full text as ‘PANK2‐LV’ with the definition ‘PKAN mutant neurons transduced with a functional copy of PANK2 by **lentiviral transduction**’. LV also appeared as ‘GFP-LV’ and ‘tdT‐LV’ later in the figure captions, indicating neurons transduced with the GFP and tdT fluorescent proteins by lentiviral transduction. Therefore, it is used to indicate a laboratory exercise instead of an organism in this article. Another example is the wild type of *Arabidopsis* (NCBI taxon: 3701), Col-0, mentioned in figure captions of the article PMC 3547818. Col-0 is the most widely used wild type of *Arabidopsis*. However, wild type information was not included in our lexicon. One possible solution is to rely on the information from the full text once again to understand that the authors used this term to refer to *Arabidopsis* wild-type plants in the Results section.

The most challenging issue for the current SPRENO implementation is that the curators of SourceData could annotate any nouns used by the authors to indicate an organism in their experiments. For instance, in the article PMC 3868461, the authors described two life cycle stages of *Trypanosoma brucei* (NCBI taxon: 5691) in their experiments, and named them as PCF (the procyclic form) and BCF (the bloodstream form) in the Introduction section. Both PCF and BCF were annotated as organism terms and linked to NCBI taxon 5691 by the curators, possibly based on the notion that both are stages in the life of *Trypanosoma brucei*. Nevertheless, they are difficult to be recognized and normalized because it required further semantic interpretation of the sentences.

### Comparison with other participating teams in the Bio-ID track

**Figure 8.**
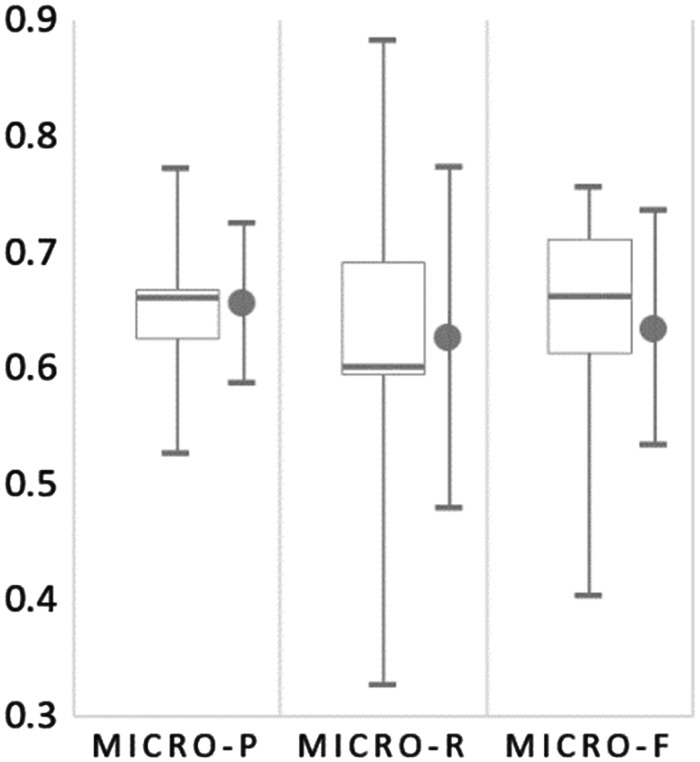
The box plot for micro-PRF scores of all submitted runs for organism normalization on the test set of the Bio-ID track. The mean and SD are shown on the right of the whiskers.

Figure 8 summarizes the normalization results for all submitted runs from the six participating teams. The extremely skewed results shown in the inter quartile for Micro-R reveal that the recall of most submitted runs in the Bio-ID track are low. The average recall is 0.626, and the only two runs with a recall over 0.7 were Run 1 and 2 submitted by our team.

On the other hand, the precision of all submitted runs were relatively higher than the recall. Two of the runs submitted by Sheng *et al.* (17) had a precision >0.7. As shown in Table 5, the micro *F*-score of their system was 0.729 with the best precision of 0.772 among all submitted runs. They constructed two models for recognizing bio-entities in their approach. One is based on the conditional random fields (CRFs) trained with standard bio-entity recognition features, and the other is a bi-directional long short-term memory (BLSTM) network in which the input words were represented by concatenating the representations of word embedding and character embedding. The word representation layer was then fed into a dropout layer followed by a BLSTM layer. The outputs of all hidden layers were shrunk to a dimension equal to the number of unique bio-entity tags in the training set using the IOBES annotation scheme, which was finally connected to a CRF layer to determine the boundaries of the bio-entities. For the normalization task, they first compiled a contextual dictionary by analysing the training set. For cases without matched IDs in the compiled dictionary, they used the NCBI Entrez web service to search the NCBI taxonomy database for candidate IDs. An entity is then linked to the known ID that shares the most contextual words with the sentence containing the entity. Although the dictionary-based approach as the one applied by this work was criticized to have a high precision but a low recall (20), we can observe that our method acquired a significantly better recall than that of Sheng *et al.* (17) in both the recognition and the normalization tasks. We attribute this to the comprehensiveness of the compiled lexicons for strain terms, common terms and the abbreviated terms observed from the full text along with the developed multistage matching algorithm, as well as the proposed common term recognition and normalization approach that may be overlooked by other teams.

The third ranked system was developed by Kaewphan *et al.* (18). Similar to our reason of implementing a new BioC reader for the task, they established a specialized tokenizer to handle some word boundary errors observed in the Bio-ID dataset. Using the tokenized dataset, they employed NER suite with customized dictionaries used in their normalization stage to train a CRF model capable of detecting all six entity types defined in the Bio-ID track. As shown in Table 4, their recognition system achieved the highest precision and *F*-scores under both matching criteria. During the normalization stage, they preprocessed terms in the NCBI taxonomy database by encoding them by *n*-gram frequencies. For each recognized organism term, they represented it as character *n*-gram frequencies and utilized the cosine similarity to calculate similarities between the term and all encoded records, and the ID with the highest cosine similarity was selected. Finally, a decision list containing heuristic rules was developed for disambiguating ambiguous organism terms. Unfortunately, their normalization approach did not perform as well as their recognition method. As demonstrated by Chang *et al.* (19), most off-the-shelf organism/species identification tools have performed poorly on the Bio-ID corpus. For example, SR4GN (4) achieved PRF-scores of 0.468, 0.382 and 0.419 on the training set, respectively. The *F*-scores of ORGANISM/SPECIES (7) on the training/test sets were also low (0.557/0.625) as exhibited by Chang *et al.* All of these tools, including the one developed by the third ranked team (18) suffered the problem of a low recall. One of the reasons may be that their normalization approaches only relied on the terms recorded in the NCBI taxonomy database, which did not cover the common terms listed in Table 2 and the terms of strain/model organisms extended in this work.

Yang *et al.* (21) also leveraged CRFs to identify organism terms. The recognized terms were normalized by heuristic rules using a dictionary compiled from the NCBI taxonomy database. To address the issue of common terms introduced in the Method section, they directly normalized these terms to the taxonomy ID with the most appearances in the article. The PRF-scores of their system were 0.609, 0.510 and 0.555, respectively. Chang *et al.* (19) studied the performance of openly available organism recognition tools on the training set of the Bio-ID track. They observed that the NCBO Annotator (22) obtained the best recall with a very low precision. In the end, they decided to adopt the ORGANISM tool developed by Pafilis *et al.* (7) and implemented a post-processing method to enhance the results. Their system achieved respective PRF-scores of 0.432, 0.580 and 0.625.

In comparison with these approaches, our method tends to acquire a better recall owing to the use of the information collected from full text. As shown in Figure 5, this strategy can significantly boost the recall of our system from 0.751 to 0.869, thereby raising the *F*-score from 0.701 to 0.732. In addition, our work serves as the pioneer in studying the feasibility of employing the CNN model for normalizing organism terms within figure captions. Currently, the advantage of applying the CNN model is imperceptible as it not only filters out certain false positive cases but also removes true positive instances. This issue may be associated to the inconsistent annotations examined in the training set and will be the direction of our future investigations.

## Conclusion

Normally, information expressed in the figure captions of published papers is extremely imperative. However, sophisticated text mining approaches are required to identify the mentioned entities and increase their discoverability. This work discloses the challenges of identifying organism terms described in figure captions by providing an analysis of the distribution of different types of organism entities observed in the figure captions. To tackle this problem, attempts were made to address the challenges including the ambiguities originated from the use of abbreviations, use of common English names that may refer to various organisms depending on the context, as well as the frequent use of strain terms with a new organism identification tool SPRENO. We demonstrated the demand of exploiting the meta-information from full text, which can greatly improve the recall and *F*-score in organism recognition and normalization. As one of the top-ranked systems in the BioCreative VI BioID track, SPRENO has exhibited its competency in recognizing and normalizing organism terms within figure captions, which may facilitate the comparison and analysis of information among various publications on the same topic.

*Conflict of interest*. None declared.

## Funding

This work was supported by the Ministry of Science and Technology of Taiwan (grant numbers MOST-105-2221-E-143-003 and MOST-106-2221-E-143-007-MY3). Funding for Open Access charge: Ministry of Science and Technology of Taiwan.
